# Linking Thyroid Function, Morphology, Autoimmunity, Body Mass Index, and Reproductive Aging to Women’s Sexual Health: Evidence from a Population Study in Kaunas

**DOI:** 10.3390/jcm14238441

**Published:** 2025-11-28

**Authors:** Dalia Daukšienė, Raimonda Klimaitė, Aistė Kondrotienė, Radvilė Matukaitienė, Jonas Čeponis, Agnė Rimkutė, Laura Dudonytė, Rasa Steponavičiūtė, Dalia Lukšienė, Vaiva Lesauskaitė, Džilda Veličkienė, Rasa Verkauskienė, Birutė Žilaitienė

**Affiliations:** 1 Institute of Endocrinology, Lithuanian University of Health Sciences, 44307 Kaunas, Lithuaniaaiste.kondrotiene@lsmuni.lt (A.K.); birute.zilaitiene@lsmu.lt (B.Ž.); 2 Medical Academy, Lithuanian University of Health Sciences, 44307 Kaunas, Lithuania; agne.rimkute1@stud.lsmu.lt (A.R.); laura.dudonyte@stud.lsmu.lt (L.D.); 3 Department of Laboratory Medicine, Lithuanian University of Health Sciences, 44307 Kaunas, Lithuania; rasa.steponaviciute@lsmu.lt; 4 Institute of Cardiology, Lithuanian University of Health Sciences, 44307 Kaunas, Lithuania

**Keywords:** sexual dysfunction, thyroid function, goiter, thyroid nodules, Hashimoto’s thyroiditis, body mass index, menopausal state

## Abstract

**Background/Objectives:** While it is known that Hashimoto’s thyroiditis (HT), goiter, thyroid nodules, and thyroid dysfunction may affect women’s reproductive health through hormonal and metabolic mechanisms, data are limited regarding the specific impacts on female sexual function. This study evaluated sexual function in women with thyroid disorders and examined its associations with thyroid function, age, menopausal status, and body mass index (BMI). **Methods:** A population-based survey was conducted in Kaunas, Lithuania, within the WHO MONICA framework. A random sample of 1569 women aged 25–69 years was included in the final analysis after applying the exclusion criteria. Anthropometric measurements were taken using standardized procedures, and the BMI was calculated. Sexual function was assessed using the 19-item Female Sexual Function Index (FSFI). Thyroid structure was evaluated by a team of trained physicians using ultrasound, while thyroid function was assessed via serum analysis (ELISA-based assays for TSH, fT4, and anti-TPO antibodies). **Results:** Of the 1569 women analyzed, 64.1% had sexual dysfunction (SD) (FSFI ≤ 26.55). Age and BMI showed significant negative correlations with all FSFI domains, with the strongest associations for arousal, lubrication, and total FSFI score (*p* < 0.01). SD was more prevalent among postmenopausal (43.6%) women than in premenopausal women (22.6%, *p* < 0.001) and increased with a higher BMI (*p* < 0.001). HT was found in 28.3% of participants. Compared with the reference group, women with HT were older, had higher BMI, higher TSH levels, and more hypothyroidism (*p* < 0.001). SD was more common in the HT group (71.7% vs. 64.2%, *p* < 0.001), with significantly lower lubrication and higher pain scores. In the multivariate analysis, only goiter remained an independent predictor of SD (*p* = 0.04). **Conclusions:** In conclusion, women with HT were older; had a higher BMI; and more frequently experienced SD, particularly reduced lubrication and increased pain, compared with the reference group. Although several thyroid conditions were associated with sexual dysfunction, only goiter remained an independent predictor after adjusting for age and BMI.

## 1. Introduction

The sexual health of adult women is an integral component of physical and psychological well-being and a determinant of quality of life, interpersonal relationships, and the overall public health burden. Sexual function in epidemiological research is most commonly measured with validated instruments such as the Female Sexual Function Index (FSFI), a multidimensional 19-item self-report scale covering desire, arousal, lubrication, orgasm, satisfaction, and pain, and is widely used in clinical and population studies [1].

Sexual function changes across the life course. Aging and the menopausal transition are consistently linked with declines in some FSFI domains, with hormonal changes, particularly falling estrogen levels, commonly implicated as biological mediators, while psychosocial and relational factors also play important roles [2,3]. However, the pattern in which FSFI domains are most affected varies between studies and populations, reflecting heterogeneity in study designs, cultural factors, and definitions of menopausal status [2].

Body composition and adiposity are other widely studied influences on female sexual function. A higher body mass index (BMI) and obesity have been associated with a higher prevalence of sexual dysfunction (SD) in many studies; however, these findings are inconsistent: some population-based longitudinal work only identified weak or domain-specific associations (for example, with desire), while other cross-sectional and meta-analytic studies reported stronger overall links between overweight/obesity and lower FSFI scores [4,5,6].

Thyroid status has emerged in the recent literature as a possible biological determinant of female sexual function. A recent meta-analysis reported lower FSFI scores among women with overt hypothyroidism and, to a lesser extent, with subclinical hypothyroidism, most consistently affecting different FSFI domains, especially lubrication, suggesting that thyroid hormones (and the metabolic and neuroendocrine changes they induce) can influence sexual physiology and experience [7]. Another meta-analysis of the sexual dysfunction in women with thyroid disorders found that the prevalence of sexual dysfunction was 59.6% (95% CI: 50.5–68.1) among those with hyperthyroidism [4]. Nevertheless, the small sample sizes in the studies and the prevalence of thyroid diseases in different regions complicate the overall understanding of the influence of thyroid function, nodules, and goiter on female sexual function.

Hashimoto’s thyroiditis represents the most common autoimmune endocrine disorder in women and has been increasingly recognized as a condition with multisystemic consequences extending beyond thyroid hormone dysregulation. Female SD is a frequent but often underappreciated comorbidity in thyroid disease [8,9,10,11]. Several studies have demonstrated that women with HT report significantly lower FSFI scores compared with the healthy reference group, even when their thyroid hormone levels are within the reference range, suggesting that factors beyond overt hypothyroidism, such as autoimmune inflammation, cytokine activity, and psychosomatic or mood-related pathways, may impair sexual health [12,13,14]. A further and unresolved question is whether thyroid autoimmunity affects sexual function independently of the measurable thyroid hormone concentrations. Some investigations indicate that euthyroid women with positive thyroid antibodies have an increased prevalence of SD and depressive symptoms, implying a possible direct autoimmune or neuroendocrine link [8,11,12,13,15]. Conversely, other studies report that after adjustment for anxiety, depression, and metabolic variables, the association between thyroid autoimmunity and SD is attenuated or lost [16]. This inconsistency highlights the need for further research into the immunological and psychosocial determinants of sexual health in women with Hashimoto’s thyroiditis, particularly in euthyroid states where clinical symptoms persist despite biochemical normalization.

In this context, we studied a population-based sample of women living in Kaunas to examine the associations between FSFI scores and key exposures: age, BMI, menopausal status, thyroid function, goiter, thyroid nodules, and thyroid autoimmunity. The aim was to estimate the independent and joint associations of these factors with overall and domain-specific sexual function. Establishing the relative contributions of metabolic, hormonal, and autoimmune pathways to female sexual function in a community sample is important both for identifying potentially modifiable targets for intervention and for informing clinicians about which patients might benefit from endocrine evaluation as part of sexual health assessment.

## 2. Materials and Methods

This epidemiological health survey of the study “Chronic Diseases and their Risk Factors in the Adult Population” (Study) was performed in Kaunas (Lithuania), following the methodology of the WHO program’s Multinational Monitoring of Trends and Determinants in Cardiovascular Disease (MONICA) study [17,18]. A random sample of males and females in Kaunas, aged 25–69 years, stratified by sex and age, was randomly selected from the Lithuanian population register (N = 6000). The call for participants started on 1 February 2020; however, due to the COVID-19 pandemic, the study was suspended on 15 March 2020, and in March 2023, the study was resumed and conducted until 21 June 2024. In total, 3426 individuals (1523 males and 1903 females) were screened. The response rate was 57.1%. The study was approved by the Kaunas Regional Ethics Committee (Lithuania) (Nr. BE-2-49; 5 June 2018). All participants provided written informed consent. For the inclusion criteria, all the selected individuals were invited.

A total of 1569 women were available for statistical analysis after excluding 334 respondents. The exclusion criteria were as follows: respondents from whom the nurses could not take a blood sample, who refused to give blood for tests, who did not fill in the questionnaires correctly, who had total thyroidectomy or hemithyroidectomy, who were pregnant, or who were using hormonal contraception or hormone replacement therapy other than thyroid hormones. 

The participants’ height was measured without footwear using a stadiometer and recorded to the nearest centimeter. The body weight was assessed to the nearest 0.1 kg using calibrated medical scales. The body mass index (BMI) was calculated as the body weight (kg) divided by the square of the height (m^2^).

The FSFI questionnaire was used to assess sexual function among the participants. The FSFI is a validated multidimensional self-report instrument consisting of 19 items that evaluate six domains of female sexual function: desire, arousal, lubrication, orgasm, satisfaction, and pain. Each domain generates a specific score, and the total FSFI score is obtained by summing the domain scores. The questionnaire has been widely used in both clinical and research settings to identify sexual dysfunction and to evaluate treatment outcomes. In this study, participants completed the FSFI based on their experiences over the preceding four weeks, following the standardized administration and scoring procedures described by Rosen et al. (2000) [1]. The total score of the FSFI ranges from 2.0 to 36.0. An optimal cut-off value of 26.55 effectively differentiates women with sexual dysfunction (SD) from those without, where higher scores reflect better sexual functioning [19]. The internal consistency of the FSFI questionnaire was assessed, revealing excellent reliability (Cronbach’s alpha = 0.917) [20].

Thyroid ultrasonography was performed by a team of trained physicians who were blinded to the clinical status of the participants. A real-time 3D/4D diagnostic ultrasound system equipped with a multifrequency transducer was used. All examinations were conducted with the subjects in a supine position and the neck in hyperextension. The thyroid volume for each lobe was calculated using the formula: width × length × thickness × 0.479. Goiter was defined as a total thyroid volume exceeding 18 mL, based on the WHO recommendations [21]. Thyroid nodules measuring more than 3 mm in diameter were recorded. According to thyroid nodularity, participants were categorized into two groups: nodule-free and those with at least one nodule.

Blood samples for the analysis of free thyroxine (FT4), thyroid-stimulating hormone (TSH), and antibodies against thyroid peroxidase (AntiTPO) were collected via venipuncture in vacuum tubes for serum with a gel separator (5 mL), following the recommendations for venous blood sampling, at room temperature. The serum samples were aliquoted and stored at −70 °C until analysis. Blood samples for the determination of FT4, TSH, and AntiTPO were analyzed via enzyme immunoassay using commercial ELISA kits (FT4 and Thyroid Peroxidase-Ab ELISA kits (TECAN, IBL International GmbH, Hamburg, Germany), a TSH ELISA kit (DIAsource, Louvain-la-Neuve, Belgium)), and an automated ELISA microplate processor Gemini (Stratec Biomedical GMBH, Birkenfeld, Germany).

Data were analyzed using IBM SPSS Statistics, version 30.0.0.0 (IBM Corp., Armonk, NY, USA). Nonparametric data were presented as the median (IQR). The normality of quantitative variables was assessed using the Kolmogorov–Smirnov test. For comparisons between two independent samples with non-normally distributed variables, the Mann–Whitney U test was applied. Categorical variables were analyzed using the chi-square (χ^2^) test. Linear relationships between continuous variables were evaluated using Spearman’s correlation analysis. A multivariate logistic regression analysis was performed to identify independent factors associated with the outcome variable. A two-tailed *p*-value ≤ 0.05 was considered statistically significant. There were missing data for some variables in the dataset. To utilize all available information without introducing bias from imputation, analyses were conducted using all available cases for each specific variable (available-case analysis). Subjects with missing values were not excluded entirely from the study but only contributed data to analyses where their values were present. Consequently, the number of cases included in each analysis varied depending on the data availability. The extent and pattern of missing data were assessed and are reported (Table 1). This approach, while retaining the sample size and avoiding assumptions required for imputation, does not address potential bias due to missingness and is a limitation of the study. Sensitivity analyses were not conducted due to the decision not to impute missing values.

## 3. Results

### 3.1. Clinical and Sociodemographic Variables

The clinical and sociodemographic characteristics of the study population are shown in Table 1. The study population consisted predominantly of middle-aged women, with a median age of 59 years (IQR: 7) and a median BMI of 27.15 kg/m^2^ (IQR: 6.86). Overall, 31.7% of participants were overweight, while 25.5% exhibited varying degrees of obesity. More than half of the women (53.4%) were postmenopausal, with a median age at menopause onset of 50 years (IQR: 6). Regarding marital status, the majority were married (62.3%), followed by divorced (14.9%).

### 3.2. Analysis of Female Sexual Function Index (FSFI) Domain Scores and Their Associations with Age and Body Mass Index

The assessment of female sexual function using the FSFI questionnaire revealed generally low scores across all domains, indicating a high prevalence of SD symptoms. Among the domains, desire and arousal showed the lowest median values, suggesting that these aspects were the most affected. Overall, 1006 participants (64.1%) had SD (FSFI ≤ 26.55). The descriptive statistics and risk percentages are summarized in Table 2.

**Figure 1:**
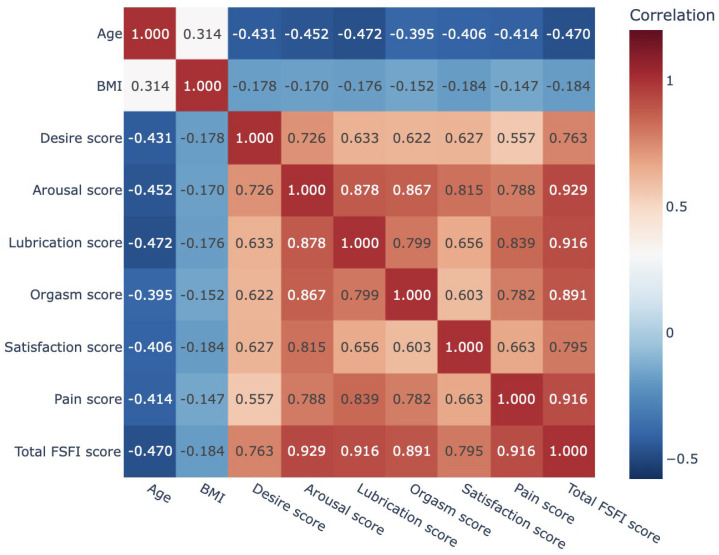
Heatmap of Spearman’s correlations for age, BMI, FSFI domains, and total FSFI score. FSFI: Female Sexual Function Index, BMI: body mass index.

Spearman’s correlation analysis revealed significant associations between age, BMI, and female sexual function parameters (Figure 1). The age showed moderate to strong negative correlations with all FSFI domains (r = −0.395 to −0.472, *p* < 0.01), indicating a decline in sexual function with increasing age. The strongest associations were observed for arousal (r = −0.452), lubrication (r = −0.472), and the total FSFI score (r = −0.470). The BMI demonstrated weak but significant negative correlations with all FSFI domains and the total score (r = −0.152 to −0.184, *p* < 0.01), with the strongest associations found for satisfaction (r = −0.184) and the total FSFI score (r = −0.184). These findings indicate that advancing age and higher BMI are associated with reduced sexual function, particularly in domains related to arousal and lubrication.

A statistically significant association was identified between menopause status and SD (χ^2^(1) = 171.262, *p* < 0.001). The prevalence of SD was substantially higher among menopausal women (43.61%) than among premenopausal women (22.57%) (*p* < 0.001). These findings indicate a strong and biologically plausible relationship between menopause and SD.

A significant association was observed between SD (FSFI ≤ 26.55) and BMI categories (*p* < 0.001). Most participants with SD were in the normal weight (24.2%) and overweight (21.7%) groups. A linear-by-linear association test confirmed a significant trend (χ^2^ = 21.76, *p* < 0.001), indicating a relationship between increasing BMI and SD.

### 3.3. Association of Thyroid Function and Morphology Characteristics with Sexual Dysfunction

Hashimoto’s thyroiditis was identified in 28.3% of subjects. Participants were divided into two groups: the HT group (women with Hashimoto’s thyroiditis) and the reference group (women without Hashimoto’s thyroiditis). The HT group was significantly older (*p* = 0.001), had a significantly higher proportion of postmenopausal women (59.7% vs. 50.9%, *p* = 0.002), and had a higher BMI (median (IQR): 27.3 vs. 25.53, *p* < 0.001). The detailed thyroid function and morphology analysis revealed that the HT group had significantly higher levels of TSH (median (IQR): 1.9 (1.47) vs. 1.41 (0.84) μIU/mL), fewer thyroid nodules (40.5% vs. 50.9%), and more hypothyroidism (5.1% vs. 0.8%) compared with the reference group (*p* < 0.028), whereas the thyroid volume, FT4 levels, and goiter prevalence did not differ significantly between the groups (Table 3).

The comparison between patients treated with thyroid hormone replacement therapy and those receiving antithyroid drugs revealed no significant differences in FSFI scores (*p* > 0.05).

The prevalence of SD (FSFI ≤ 26.55) was statistically significantly higher in the HT group compared with the reference group (71.7% vs. 64.2%, *p* < 0.001). The HT group had a significantly lower lubrication score compared with the reference group (*p* = 0.019). The score for the pain domain was also significantly lower in the HT group than in the reference group (*p* = 0.046). No statistically significant differences were found between the groups for the desire, arousal, orgasm, or satisfaction domains. Furthermore, although the total score was lower in the HT group than in the reference group, this difference did not reach statistical significance (Table 4). The comparison between patients treated with thyroid hormone replacement therapy and those receiving antithyroid drugs revealed no significant differences in the FSFI scores (*p* > 0.05).

We evaluated the association between various thyroid factors and SD (FSFI ≤ 26.55). All three thyroid conditions (HT, goiter, and thyroid nodules) emerged as significant predictors of SD. HT had 1.42 times higher odds of SD (*p* = 0.005), the presence of goiter was linked to a 2.16-fold increase in the odds of SD (*p* = 0.004), and thyroid nodules were associated with 1.32 times the odds of SD (*p* = 0.012) (Table 5).

Multivariate logistic regression analysis revealed that after adjusting for age and BMI, only goiter remained a statistically significant independent predictor for SD (*p* = 0.04), while the associations with HT and thyroid nodules were attenuated to non-significance (Table 6).

## 4. Discussion

In this large cross-sectional study involving 1569 women, we observed that those with HT demonstrated modest but significant impairments in SD compared with the reference group. Specifically, women with HT had lower FSFI scores in the lubrication and pain domains, suggesting that autoimmune thyroid disease may adversely affect these aspects of sexual response and comfort. Overall, more than sixty percent of the participants had SD, with menopausal status, age, and BMI emerging as significant determinants. Postmenopausal women and those with higher BMI values exhibited a higher prevalence of SD, underscoring the multifactorial nature of this condition. Clinically, women with HT also presented with a higher BMI, higher obesity prevalence, and a higher proportion of menopausal status, and thyroid function abnormalities were more common than in the reference group. Although several thyroid conditions (goiter, thyroid nodules, and HT) were associated with SD, the multivariate logistic regression analysis showed that, after adjusting for age and BMI, only goiter remained a statistically significant independent predictor of SD.

The strengths of this epidemiological study lie in its comprehensive population-based design and the multidimensional assessment of women’s sexual health in relation to thyroid function, autoimmunity, body mass index, and aging. By including a well-characterized cohort from Kaunas, the study benefits from detailed clinical, biochemical, and hormonal profiling, which allows for the robust exploration of complex endocrine and reproductive associations. The use of standardized measures for thyroid parameters and the FSFI questionnaire enhances the data reliability and comparability with other populations. Moreover, the integration of autoimmune markers provides novel insights into potential immune–endocrine mechanisms underlying sexual health disturbances. The population-based sampling also reduces selection bias, improving the generalizability of findings to 25–69-year-old women living in Kaunas, Lithuania.

The main weaknesses of this study stem from its cross-sectional design, which limits the ability to infer causal relationships between thyroid function, autoimmunity, body composition, reproductive aging, and sexual health. As data were collected at a single time point, temporal dynamics and potential bidirectional interactions cannot be fully captured. Self-reported information on sexual health may also introduce reporting bias due to social desirability or recall limitations, particularly in sensitive domains such as sexual function. Additionally, while the study included key hormonal and immunological parameters, it did not incorporate standardized and validated psychometric instruments—such as the Beck Depression Inventory for depressive symptoms [22], the State–Trait Anxiety Inventory for anxiety [23], the Perceived Stress Scale for stress [24], or the Dyadic Adjustment Scale for relationship satisfaction [25]—which limits our ability to fully account for the psychological and relational factors that may influence female sexual function and confound the observed associations. Furthermore, while age adjustment partially controls for menopausal status due to its strong link, we acknowledge that residual confounding from postmenopausal hormonal changes may still exist. The use of available-case analysis, while maximizing data use without imputation, may have introduced bias due to missing data and variable sample sizes across analyses. The study sample, although population-based, was geographically limited to Kaunas, which may restrict the generalizability of findings to other ethnic or cultural populations. Finally, the relatively small number of participants with thyroid dysfunction may have reduced the statistical power to detect subtle subgroup differences.

We observed that increasing age among women was associated with lower FSFI scores. Our findings are consistent with several cross-sectional studies demonstrating a negative correlation between age and total FSFI score, suggesting a decline in overall sexual function with advancing age [26,27]. However, not all studies have reported similar results. Sato et al. investigated women aged 40–79 years and found significant age-related decreases in sexual desire, arousal, and lubrication, along with increased pain during intercourse; however, the orgasm and satisfaction scores did not significantly decline with age in their cohort [6]. In contrast, Javadifar et al. reported no significant association between age and SD, although their study population was limited to women under 45 years of age [28]. Differences in age distribution and menopausal status across studies, particularly between reproductive-age and peri-/postmenopausal samples, likely contribute to the observed variability in results. A key strength of our research is the inclusion of a broad age range of women (25–69 years), allowing for a more comprehensive assessment of age-related variations in sexual function across both reproductive and postmenopausal stages.

A statistically significant relationship was observed between menopausal status and SD, with postmenopausal women exhibiting a markedly higher prevalence of SD compared with premenopausal women in our study. These results are in line with most recent cross-sectional studies [26,28,29,30,31,32], as well as a systematic review by Heidari et al. [2], which shows that the prevalence of SD increases substantially during and after menopause, largely due to hormonal fluctuations. Heidari et al. also highlighted that psychosocial and physiological factors, such as mood disturbances, sleep disorders, and body image changes, further exacerbate SD in postmenopausal women [2]. However, it should be noted that a limitation of our study is the lack of assessment of psychological factors that may contribute to SD. Variables such as mood, anxiety, relationship satisfaction, and stress levels were not evaluated, which may have provided a more comprehensive understanding of the multifactorial nature of SD in women. Therefore, our findings primarily reflect the association between physiological factors and SD, while the potential influence of psychological determinants remains to be explored in future research.

Jonusiene et al. analyzed sexual function in a clinical sample of Lithuanian postmenopausal women. They identified the most important determinants of sexual function, including the use of hormone replacement therapy, emotional status, and menopausal symptoms. Sexual function was better in younger women and in hormone replacement therapy users compared with non-users in this study [3]. In our study, we excluded women who were using hormone replacement therapy or hormonal contraception. This exclusion may be considered a limitation, as it reduces the generalizability of the findings to the broader population of women, many of whom use hormonal therapy. Consequently, our results may not fully reflect the diversity of hormonal and clinical profiles seen in real-world settings, and the exclusion may introduce selection bias if hormonal treatment users differ systematically from non-users regarding health status, symptom severity, or lifestyle factors. However, we believe this approach can also be regarded as a strength, as it minimizes the confounding effect of exogenous menopausal hormones on sexual function and allows for a more accurate assessment of the natural associations between endogenous hormonal changes, menopausal symptoms, and sexual function. By focusing on women not using menopausal hormonal treatments, our study provides a clearer picture of the physiological determinants of sexual function in the natural postmenopausal state.

In the present study, a higher BMI was associated with a modest reduction in sexual desire and overall sexual function, consistent with prior evidence linking obesity to impaired sexual health in women [33]. The probability of sexual dysfunction increased progressively across obesity categories, aligning with the findings of Faubion et al. [34] and the systematic review by Salari et al. [4], both of which demonstrated similar trends of deteriorating sexual function with greater adiposity. Notably, the relatively high prevalence of SD observed among underweight women by Logan et al. [35] suggests that deviations from normal body weight in either direction (obesity or underweight) may adversely affect hormonal regulation and body image, thereby compromising sexual well-being. Collectively, these observations underscore the importance of maintaining a healthy body weight as a potential factor in preserving optimal female sexual function.

The issue we tried to address with this population-based study was the impact of thyroid autoimmunity on SD. We found that women with HT showed slightly lower FSFI scores across all domains compared with the reference group. Statistically significant differences were noted in the lubrication and pain domains, indicating diminished sexual function in these areas among participants with HT compared with those without this autoimmune condition. Several previous studies have reported similar findings, supporting the notion that thyroid autoimmunity may negatively influence female sexual function, even in the euthyroid state. For instance, Bortun et al. [8] and Krysiak et al. [12] observed lower total FSFI scores among women with HT compared with the healthy reference group, suggesting that autoimmune-related mechanisms, rather than thyroid hormone levels alone, may contribute to SD. In particular, impaired lubrication and increased pain during intercourse have been consistently described, potentially linked to subtle alterations in sex hormone metabolism, endothelial function, and inflammatory cytokine activity associated with chronic autoimmune processes. Conversely, some authors, such as Lou et al. [16], did not find significant differences in overall FSFI scores between euthyroid women with HT and a reference group, emphasizing that psychological and psychosocial factors, such as anxiety, fatigue, or body image concerns, may mediate rather than directly result from the autoimmune condition. Our results, showing domain-specific reductions in lubrication and pain, but only modest overall FSFI changes, may therefore represent an intermediate pattern, highlighting that thyroid autoimmunity can exert selective effects on sexual function components. This finding aligns with the growing understanding that autoimmune inflammation and its systemic effects may impair vascular and mucosal responses and reduce sexual comfort and arousal, particularly in women with long-standing HT [8,13]. Several possible mechanisms have been proposed. One mechanism involves systemic inflammation: HT is characterized by immune-mediated lymphocytic infiltration of the thyroid and elevations of anti-thyroid autoantibodies, and in euthyroid women, the severity of SD correlates with levels of antibodies and inflammatory markers (high-sensitivity C-reactive protein) rather than just TSH/FT4 [13]. A second mechanism pertains to alterations in sex steroid metabolism: studies in euthyroid HT women found lower free testosterone (or free androgen index) and associations between testosterone levels and domains of libido and arousal, suggesting autoimmune thyroid disease may reduce androgen bioavailability or production (for example, via adrenal zona reticularis dysfunction or DHEA metabolism) even in the absence of frank thyroid hormone abnormality [13]. A third mechanism is central nervous system/psychological: autoimmunity, which may affect brain excitability or autonomic regulation, leading to decreased libido independent of thyroid hormone levels; indeed, altered central excitability was observed in HT independent of TSH, and depressive symptoms (more frequent in HT) correlate with worse sexual function (especially lubrication and satisfaction domains) in euthyroid patients [13]. In sum, HT may promote SD via inflammatory, sex-hormone-related, and neuro-psychological pathways, even when the standard thyroid function tests are within normal limits.

However, the primary finding of this study is that goiter remained an independent and statistically significant predictor of SD after adjusting for age and BMI (adjusted OR = 1.82; *p* = 0.04). This suggests that the morphological presence of a goiter, rather than just the autoimmune status or nodules, has a distinct association with female sexual function. Pasqali et al. showed that nodular goiter was linked to the highest prevalence of female SD (68.4%), suggesting that even euthyroid structural thyroid abnormalities can influence sexual health [10]. Several potential mechanisms may explain our finding that goiter independently predicts female SD. Goiter could be a clinical marker for iodine deficiency, which impacts hormone synthesis and sexual function even with normal TSH. This hypothesis is strongly supported by recent epidemiological data: a 2022 nationwide study of the Lithuanian adult population found the median urinary iodine concentration to be 95.5 µg/L [35], which is below the 100 µg/L threshold defined by the World Health Organization as sufficient, placing the population in a state of mild iodine deficiency [36]. Conversely, in iodine-sufficient populations, goiter is more often a sign of HT, where chronic inflammation and not goiter is the driving factor. Lacking data on iodine status, our study could not distinguish these pathways, highlighting a limitation. The other possible mechanism is that a large or visible goiter can have a significant psychosocial impact, negatively affecting body image, self-esteem, and mood [33], all of which are critical components of sexual health. Compressive symptoms from a large goiter (e.g., discomfort, dysphagia) could contribute to a general decline in physical well-being [37,38], thereby impacting sexual desire and function. Furthermore, goiter may be a proxy for a longer duration or higher severity of thyroid disease not fully captured by TSH levels alone [39]. The persistence of this association after adjustment suggests that structural or hormonal alterations associated with goiter may contribute to disturbances in sexual health. These results highlight the importance of evaluating thyroid morphology and function in the assessment of SD among women, as addressing underlying thyroid abnormalities could potentially improve sexual well-being and overall quality of life. Routine assessment of sexual health (using validated tools such as the FSFI) should be integrated into patient evaluation. Associations with menopausal status and BMI emphasize a multidisciplinary approach addressing endocrine, metabolic, and reproductive factors. Optimizing thyroid function, managing weight, and controlling menopausal symptoms may improve sexual well-being [40]. Open discussions in thyroid clinics can help reduce the stigma and facilitate timely, comprehensive care.

Building on these findings, future research should focus on longitudinal and mechanistic studies to clarify causal relationships between thyroid function and nodules, goiter, HT, body composition, menopausal status, and SD. Prospective cohort studies could track sexual function over time in women with autoimmune thyroid disease, distinguishing between euthyroid and hypothyroid states, to determine whether changes in thyroid autoimmunity precede or follow declines in sexual function. Integrating psychological, relational, and lifestyle factors into future studies will provide a more holistic understanding of female sexual health in this population. Finally, interventional trials assessing whether targeted treatments, such as weight management, hormonal supplementation, or anti-inflammatory strategies, can mitigate SD in women with Hashimoto’s thyroiditis would offer clinically actionable insights and inform personalized management approaches.

To conclude, the present study finds that age, menopause, and higher BMI are associated with a lower FSFI score. Women with HT experienced disturbances in sexual function, mainly involving dysfunction in lubrication and pain. Although multiple thyroid disorders (goiter, thyroid nodules, and HT) were linked to SD, only goiter remained an independent predictor after adjusting for age and BMI. The findings add population-level evidence to ongoing debates about endocrine and metabolic determinants of women’s sexual health and may help guide targeted clinical assessment and future research priorities.

## Figures and Tables

**Table 1:** Characteristics of the study population (n = 1569).

Variable	Category/Description	n	%	Median (IQR)
Age (years)				59 (7)
Age at menopause onset (years)				50 (6)
BMI (kg/m^2^) BMI categories				27.15 (6.86)
	Underweight	29	1.8	
	Normal weight	642	40.9	
	Overweight	498	31.7	
	Class I obesity	262	16.7	
	Class II obesity	104	6.6	
	Class III obesity	34	2.2	
Menopause status	Menopausal	824	53.4	
	Premenopausal	720	46.6	
Marital status	Married	975	62.3	
	Single	168	10.7	
	Divorced	233	14.9	
	Cohabiting	86	6.6	
	Widowed	103	5.5	
Thyroid function	Euthyroidism	1223	96.9	
	Hypothyroidism	25	2.0	
	Hyperthyroidism	14	1.1	
Thyroid morphology	Nodule(s)	748	49.9	
	No nodule(s)	752	50.1	
Autoimmunity	HT	444	28.4	
	no HT	1117	71.6	

Median (IQR), BMI: body mass index, HT: Hashimoto’s thyroiditis.

**Table 2:** Female Sexual Function Index (FSFI) domain scores.

FSFI Domain	Median (IQR)	SD
Desire	3.00 (1.8)	-
Arousal	3.30 (3.9)	-
Lubrication	4.20 (5.7)	-
Orgasm	3.60 (5.2)	-
Satisfaction	4.00 (3.2)	-
Pain	4.40 (6.0)	-
Total FSFI	23.30 (24)	64.10%

Median (IQR), SD: sexual dysfunction, FSFI: Female Sexual Function Index.

**Table 3:** Thyroid function and morphology characteristics in patients with Hashimoto’s thyroiditis and the reference group.

Characteristic	HT Group (n = 444)	Reference Group (n = 1117)	*p*-Value
Age (years)	54 (18)	51 (19)	0.001
Menopause (yes), n (%)	262 (59.7)	559 (50.9)	0.002
BMI (kg/m^2^)	27.3 (7.82)	25.53 (6.97)	<0.001
Thyroid volume (mL)	8.6 (5.82)	8.71 (5.67)	0.455
TSH (μIU/mL)	1.9 (1.47)	1.41 (0.84)	<0.001
FT4 (ng/dL)	1.0 (0.21)	0.98 (0.2)	0.183
Goiter n (%)	31 (7.2)	64 (6.0)	0.377
Thyroid nodules n (%)	180 (40.5)	568 (50.9)	<0.001
Thyroid function:			
Euthyroidism, n (%)	332 (93.5)	891 (98.2)	<0.001
Hypothyroidism, n (%)	18 (5.1)	7 (0.8)	0.028
Hyperthyroidism, n (%)	5 (1.4)	9 (1.0)	0.285

Median (IQR), BMI: body mass index, FT4: free thyroxine, TSH: thyroid-stimulating hormone, HT: Hashimoto’s thyroiditis.

**Table 4:** Female Sexual Function Index domain scores and sexual dysfunction in women with and without Hashimoto’s thyroiditis.

FSFI Domain	HT Group (n = 444)	Reference Group (n = 1117)	Total n = 1561	*p*-Value
Desire	3.0 (1.8)	3.0 (1.8)	3.0 (1.8)	0.119
Arousal	3.3 (3.6)	3.3 (3.9)	3.3 (3.9)	0.100
Lubrication	3.9 (5.4)	4.2 (5.7)	4.2 (5.7)	0.019
Orgasm	3.6 (4.8)	3.6 (5.2)	3.6 (5.2)	0.397
Satisfaction	4.0 (3.0)	4.0 (3.2)	4.0 (3.2)	0.414
Pain	4.0 (6.0)	4.8 (6.0)	4.4 (6.0)	0.046
Total FSFI	21.5 (24)	23.1 (25)	22.5 (24)	0.076
SD (FSFI ≤ 26.55) n (%)	312 (71.7)	693 (64.2)	1006 (64.1)	<0.001

Median (IQR), SD: sexual dysfunction, FSFI: Female Sexual Function Index, HT: Hashimoto’s thyroiditis.

**Table 5:** Association of thyroid factors with sexual dysfunction.

Thyroid Factor		SD (+) (FSFI ≤ 26.55) n (%)	SD (−) (FSFI > 26.55) n (%)	*p*-Value	OR (95% CI)
HT	Yes No	312 (71.7) 693 (64.2)	123 (28.3) 387 (35.8)	0.005	1.42 (1.11–1.81)
Goiter	Yes No	73 (80.2) 911 (65.3)	18 (19.8) 484 (34.7)	0.004	2.16 (1.27–3.65)
Thyroid nodules	Yes No	517 (69.4) 489 (63.3)	228 (30.6) 284 (36.7)	0.012	1.32 (1.06–1.63)

FSFI: Female Sexual Function Index, SD: sexual dysfunction; HT: Hashimoto’s thyroiditis.

**Table 6:** Multivariate logistic regression analysis of thyroid factors for sexual dysfunction.

Risk Factor	Unadjusted OR (95% CI) *	*p*-Value	Adjusted OR (95% CI) *	*p*-Value
HT	1.5 (1.17–1.92)	0.001	1.29 (0.98–1.68)	0.065
Goiter	2.0 (1.17–3.38)	0.011	1.82 (1.03–3.2)	0.040
Thyroid nodules	1.35 (1.09–1.69)	0.007	0.81 (0.63–1.04)	0.094

* Adjusted for age and BMI; HT: Hashimoto’s thyroiditis.

## Data Availability

The original contributions presented in this study are included in the article. Further inquiries can be directed to the corresponding author.

## Abbreviations

The following abbreviations are used in this manuscript:

Anti-TPO	anti-thyroid peroxidase antibodies
HT	Hashimoto’s thyroiditis
BMI	body mass index
FSFI	Female Sexual Function Index
fT4	free thyroxine
SD	sexual dysfunction
TSH	thyroid-stimulating hormone
